# Acknowledgment to Reviewers of *Journal of Functional Morphology and Kinesiology* in 2021

**DOI:** 10.3390/jfmk7010015

**Published:** 2022-01-26

**Authors:** Journal of Functional Morphology Kinesiology Editorial Office

**Affiliations:** MDPI AG, St. Alban-Anlage 66, 4052 Basel, Switzerland

Rigorous peer-reviews are the basis of high-quality academic publishing. Thanks to the great efforts of our reviewers, *JFMK* was able to maintain its standards for the high quality of its published papers. Thanks to the contribution of our reviewers, in 2021, the median time to first decision was 19 days and the median time to publication was 37 days. The editors would like to extend their gratitude and recognition to the following reviewers for their precious time and dedication, regardless of whether the papers they reviewed were finally published:
Abdul Rashid AzizJoohyung LeeAdam GonzalezJosé Afonso NevesAdam SchifferJosé Miguel Martínez-SanzAdam WellsJozef SimenkoAdriano PiattelliJulia BujanAleksandra FilipJulio CostaAleksandra ŻebrowskaKatsuhiko SuzukiAlessandro De SireKe-Vin ChangAlessio TorcinaroKijin KimAlex Buoite StellaKil-Soo KimAndersen JordanKok Yong ChinAndrea FuscoKonstantinos PapadimitriouAndrew LaneKonstantinos PapanikolaouAndrew LavenderKonstantinos SidiropoulosAngeliki-Nikoletta StasinakiKoos Jaap Van ZwietenAntonio CicchellaKoutlianos NikolaosAntonio PalmaKrzysztof ChmielowiecArkadiusz StanulaKrzysztof KusyArne KienzleLeandro José Rodrigues MachadoAthos TrecrociLeonardo Alexandre Peyré-TartarugaAurelia Beatrice AbalaseiLuigi-alberto PiniBartłomiej NiespodzińskiMansour GhasemikaramBeata TarnackaMarcin MaciejczykBerger NinaMarco GovoniBeth LeeMarcos Gutiérrez-DávilaBritton W. BrewerMaria PaparoupaCarla LoretoMaria Teresa TomasCatherine Van Der StraetenMarianne HuebnerChariklia K. DeliMarília Santos AndradeChe-Yu LinMarina QuartuChristopher BallmannMarinella CocoChristopher LatellaMario VetranoChristos K. YiannakopoulosMariola PawlaczykChung-Hwan ChenMark Elisabeth Theodorus WillemsCristina Castejón-RiberMarkus TilpCristina CortisMateusz KuklaDagmar PavluMatteo BalzarroDana BadauMatthias GatzDanuta UmiastowskaMichael H. StoneDavid BerryMichael J. JurynecDavid FindlayMichael StoneDavid O'SullivanMichele VecchioDiego MinciacchiMiguel Ángel Rojo TiradoDonatella Di CorradoMike McGuiganDouglas DunlopMohd Parvez KhanEdith Van DyckMoisés MarquinaEleftherios KellisNai-Jen ChangElena Mainer PardosNemanja LakicevicElena TsourdiNicola MarottaEleuterio Sanchez RomeroNobuaki HamazakiEliška SovováOliver LudwigEllen KemlerPaulo GentilElse Marie BartelsPedro Sáenz-LopezEnrique NavarroPeter HofmannErica MenegattiPetr StastnyErik CattryssePhilippe Von RothEvan P. PashaPietro CacialliFelice SiricoPietro PicernoFranca NatalePiotr GawdaFrancesca LatinoPiotr MorasiewiczFrancesco CampaR. Andrew ShanelyFrancesco FischettiRachael K. NelsonFrancisco Pradas De La FuenteRaquel Leirós-RodríguezGaia PalminiRaul DomínguezGiacomo RossettiniRazvan DragoiGiuseppe CoratellaReidar P. LystadGiuseppe MusumeciRené R.H. HartensuerGonzalo MarquezRichard JenkinsonGrégoire CattanRobert F. BentleyHadi NobariRobert W. MoellerHamdi ChtourouRoberto CannataroHelder Miguel FernandesRobin SouronHenk KoppelaarRong-Sen YangHenrique NascimentoRui NevesHisayoshi OkamuraSandra ZampieriHrvoje KarninčićSantiago ZabaloyIgnacio RefoyoSean MaloneyIker MadinabeitiaSergey MalkinImmacolata BelvisoShin-Tsu ChangImmacolata Cristina NettoreSimon RinaldiIonela Ruxandra SfeatcuSonia J. Romero MartínezIrene AprileSouhail HermassiJames PethickSreenivasan JayakumarJared GollieStefano PalermiJavier Courel IbáñezStephen CornishJeffrey RothschildThayse Natacha GomesJihong ParkThomas NikodelisJin-Hwa JungThomas StögglJiří SuchýVassilios PanoutsakopoulosJoão Paulo BritoVetrano MarioJonida HaxhiVirginia TancrediJoo Young KimVonk Lucienne

